# Synthesis, CP-MAS NMR Characterization, and Antibacterial Activities of Glycine and Histidine Complexes of Cd(SeCN)_**2**_ and Hg(SeCN)_**2**_

**DOI:** 10.1155/2013/476874

**Published:** 2013-02-28

**Authors:** Bassem A. Al-Maythalony, M. Monim-ul-Mehboob, Mohammed I. M. Wazeer, Anvarhusein A. Isab, M. Nasiruzzaman Shaikh, Saleh Altuwaijri

**Affiliations:** ^1^Department of Chemistry, King Fahd University of Petroleum and Minerals, Dhahran 31261, Saudi Arabia; ^2^Center of Research Excellence in Nanotechnology (CENT), King Fahd University of Petroleum and Minerals, Dhahran 31261, Saudi Arabia; ^3^Clinical Research Laboratory, Saad Research & Development Center, Saad Specialist Hospital, Al-Khobar 31952, Saudi Arabia

## Abstract

The synthesis and characterization of cadmium and mercury complexes of selenocyanate of the type [(L)M(SeCN)_2_] are described, where L is L-Histidine (His) or L-Glycine (Gly) and M is Cd^2+^ or Hg^2+^. These complexes are obtained by the reaction of 1 equivalent of respective amino acids with metal diselenocyanate precursor in a mixture of solvents (methanol : water = 1 : 1). These synthesized compounds are characterized by analytical and various spectroscopic techniques such as elemental analysis (EA), IR, H,1 and C13 NMR in solution and in the solid state for C13 and N15. The *in vitro* antibacterial activities of these complexes have been investigated with standard type cultures of *Escherichia coli* (MTCC 443), *Klebsiella pneumoniae* (MTCC 109), *Pseudomonas aeruginosa* (MTCC 1688), *Salmonella typhi* (MTCC 733), and *Staphylococcus aureus* (MTCC 737).

## 1. Introduction

Since the metal ions play vital roles in a number of biological processes such as biomolecules stabilizations, enzyme regulations, transportation of fluids through transmembrane channels, and so forth [1–3], numerous metal ions amino acids complexes also act as potent antifungal, antibacterial, and anticancer drugs [4–6]. Therefore, the extensive studies of these metallic species have been dedicated to understand the impact on living systems. It is well known that metal-binding proteins cover a large fraction of the total protein, and they are actively participating in several essential life processes [2, 3]. Therefore, understanding of the physicochemical and biochemical properties of metals with amino acids becomes indispensible and a broad area of research for several years [7–11].

Among all amino acids found in nature, Histidine is often found as a ligand in various types of metalloenzymes because it is the key amino acids residue in many enzymatic reactions [12]. This is may be due to its stereochemical location of the coordinating atom in Histidine. The Histidine skeleton contains the imidazole side group having two nitrogen atoms capable of participating in metal-ligand coordination sphere thereby it can take on various metal-bound forms in proteins. Thus, it is important to know the coordination modes of the Histidine (His) and Glycine (Gly) ligands to understand the reaction mechanism of metalloenzymes [13].

The coordination modes of various metal ions with amino acids have been the topic of discussion for a long period of time, and the ideas to get the binding modes are not easy to predict for amino acids with large side chain such as Histidine [16], because of different types of donor atoms present in amino acid backbone. In this point it has become necessary to study its active sites and binding affinity to transition metals at both theoretical and experimental levels. In line with efforts made by theoretical studies it has appeared that the stereochemical suitability of the metal ions play a critical role in determining the location of bond formations [17].

Selenocyanate ligand can have versatile binding modes [18, 19]; nevertheless soft Se center is expected to coordinate more preferably to soft metals leaving the harder N uncoordinated [20, 21].

In order to gain better understanding of the interaction of the metal ions with macromolecules involving amino acids, knowledge of the structure and the energetic of the metal ions coordination to amino acids are required. In an effort to obtain a more complete picture, we have synthesized a number of hitherto unknown cadmium and mercury selenocyanate complexes and their characterization using various important spectroscopic techniques.

## 2. Experimental

### 2.1. General Remarks

### 2.2. Preparation of Cd(II) and Hg(II) Complexes

A solution of CdCl_2_ in 10 mL dist. water was mixed with a stoichiometrically equivalent amount of ligand (Histidine or Glycine) in 10 mL solvent mixture (methanol : water = 1 : 1 in volume), produced solution stirred for 30 min, then two equivalents KSeCN water solution was added, the resulting mixture fluxed with nitrogen gas with stirring for 15 min then heat it for ~1.5 hour at 70°C. The product was filtered and dried. The same procedure was applied for mercury complexes using HgCl_2_ instead of CdCl_2_.

### 2.3. Spectroscopic Measurements

The measurements of solid-state IR and solution NMR were recorded as described in the literature [22, 23]. The solution NMR chemical shifts of ligands along with corresponding complexes are given in Tables 3 and 4.

### 2.4. Solid-State NMR Studies

Natural abundance ^13^C solid-state NMR spectra were obtained on a JEOL LAMBDA 500 spectrometer operating at 110.85 MHz, corresponding to a magnetic field of 11.74 T, at ambient temperature of 25°C. Samples were packed into 6 mm zirconia rotors. Cross-polarization and high power decoupling were employed. Pulse delay of 7.0 s and a contact time of 5.0 ms were used for carbon observations in the CPMAS experiments, whereas the pulse delay of 10 s and a contact time of 6.0 ms were used in the selenium observation. The magic angle spinning rates were from 3000 to 5000 Hz. ^13^C chemical shifts were referenced to TMS by setting the high-frequency isotropic peak of solid adamantane to 38.48 ppm.

The ^15^N NMR spectrum was recorded at 50.55 MHz using ^15^NH_4_ NO_3_ as external reference, which lies at −358.62 ppm relative to pure MeNO_2_ [24]. The spectral conditions for ^15^N were 32 K data points, 0.721 sec acquisition time, 2.50 sec delay time, 60° pulse angle, and approximately 5000 scans. The chemical shift of nitrogen was initially referenced with respect to liquid NH_3_, by setting the ^15^N peak in enriched solid ^15^NH_4_Cl to 40.73 ppm [25] and then converted to the standard nitromethane by a shift of −380.0 ppm [24] for ammonia. The ^13^C and ^15^N spectra containing spinning side-band manifolds were analyzed using a computer program WSOLIDS developed at Dalhousie and Tubingen universities [26].

### 2.5. Computational Study

Geometry optimization was done for the built structures and optimized by DFT level of theory with LanL2DZ (Los Alamos ECP plus double zeta) [27, 28] basis sets using Gaussian 09, Revision A. 1 program package [29].

### 2.6. Test of Bacterial Strains

Standard type cultures of *Escherichia coli* (MTCC 443), *Klebsiella pneumoniae* (MTCC 109), *Pseudomonas aeruginosa* (MTCC 1688), *Salmonella typhi* (MTCC 733), and *Staphylococcus aureus* (MTCC 737) were obtained from Microbial Type Culture Collection (MTCC) Chandigarh, India). The agar well-diffusion technique [30] was used to screen the antibacterial activity. *In vitro* antibacterial activities were screened by using nutrient agar plates obtained from HiMedia (Mumbai, India). The plates were prepared by pouring 20 mL of molten media into a sterile Petri dish and allowed to solidify for 5 minutes (Table 1). A sterile cork borer of diameter 6.0 mm was used to make wells in the agar plates. Inoculums were swabbed uniformly on the surface of agar plates. 0.1 mg/well were loaded on 6.00 mm diameter wells. The plates were allowed to stand for 1 h for diffusion then incubated at 37°C for 24 hrs. At the end of incubation, inhibition zones were measured.

## 3. Results and Discussions

### 3.1. IR and NMR Studies

The ^13^C solution NMR data of all complexes were shown in Table 3. The downfield chemical shifts were observed for the prepared complexes for (Gly)Cd(SeCN)_2_ at 194.9 ppm and (Gly)Hg(SeCN)_2_ at 189.24 with respect to the free ligand, Glycine at 173.1 ppm. These high downfield shifts resulted from the electron donation from Glycine carboxylate to metal thereby causing about 20 ppm shifts of carbonyl carbon, while this shift was not observed in the Histidine complexes because, in Histidine complexes, imidazole nitrogen and *α*-amine are involved in coordination to the metal center, which agree with the reported binding mode of Histidine to mercury metal ion as shown in Figure 3 [31].

The C*≡*N infrared frequency for Hg(SeCN)_2_ is higher than that for Cd(SeCN)_2_ which means stronger C–N bond, and this lead to less electron density at the selenium atom that derives more back donation from the Hg to Se, which makes Hg–Se bond stronger. This less electron density observed also by downfield shift for the de-shielded Se bound to Hg (−109 ppm) compared with Se bound to Cd (−272 ppm) as shown by ^77^Se NMR (Table 4). In case of Histidine complexes of Hg(SeCN)_2_ selenium atom became more shielded and shifted upfield (−169.71 ppm) because of donation from Histidine to the metal center, which causes even stronger *π*-back donation to selenium. In Table 2, the IR data shows the highest red shift for selenocyanate frequency for Histidine complex of mercury which means the highest *π*-back donation from the metal to the selenocyanate rather than Glycine so greater donation to the antibonding *π*-orbitals of the cyanate from selenium atom indication of stronger Histidine mercury bonding than the rest of the complexes series. This is also clear from ^77^Se NMR data, which showed greater deshielding effect at the selenium via complexing to Histidine, which binds through two nitrogen atoms [32]. In general, good agreement of the experimental and theoretical IR starching bands observed for the prepared complexes with some blue shift of the calculated results because of the intermolecular interaction in the real IR experiment.

**Figure 1:**
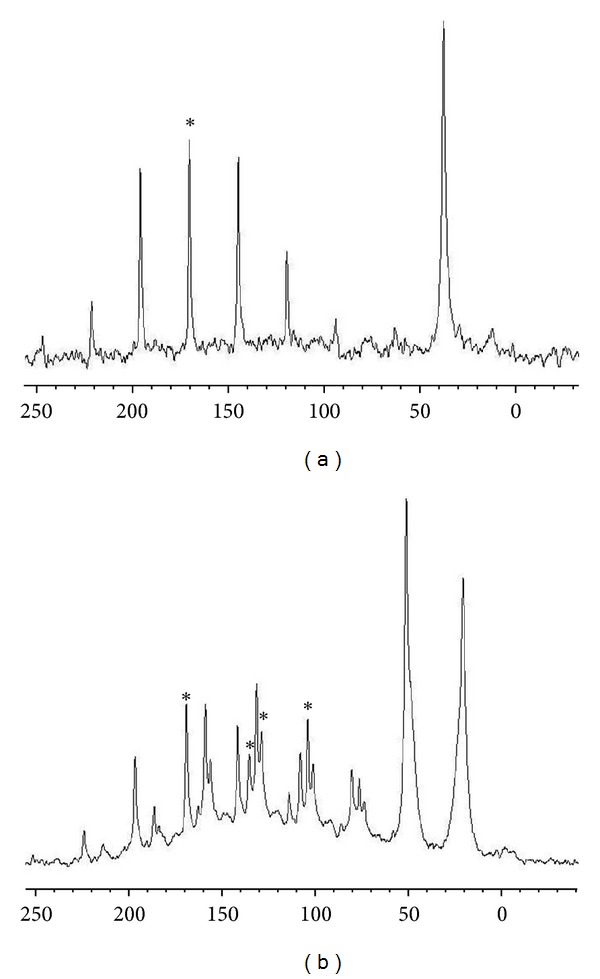
^13^C CPMAS spectra of (a) (Gly)Cd(SeCN)_2_, (b) (His)Cd(SeCN)_2_. The center peak is denoted by “∗.”

**Figure 2:**
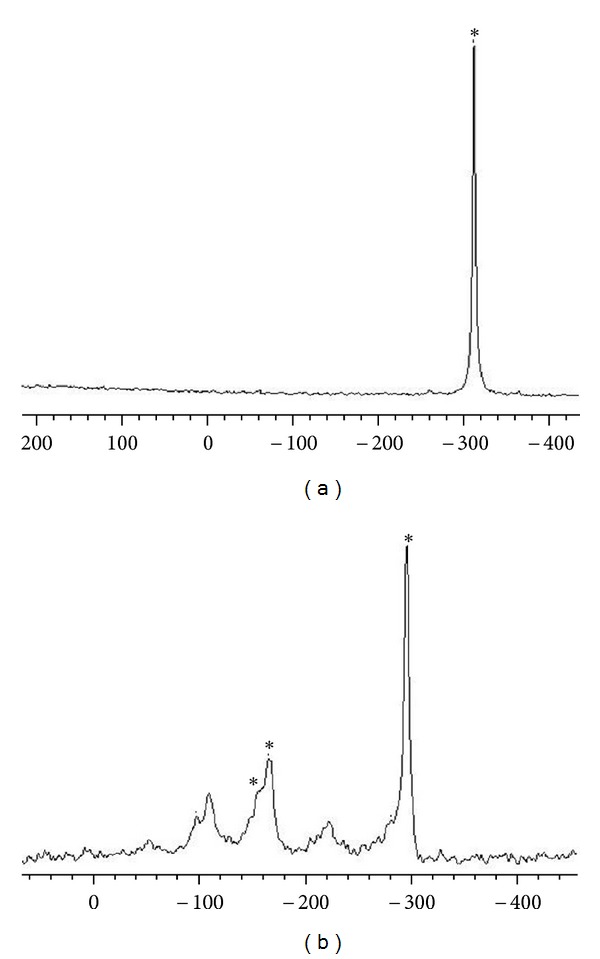
^15^N NMR spectra of (a) (Gly)Hg(SeCN)_2_ and (b) (His)Hg(SeCN)_2_.

**Figure 3:**
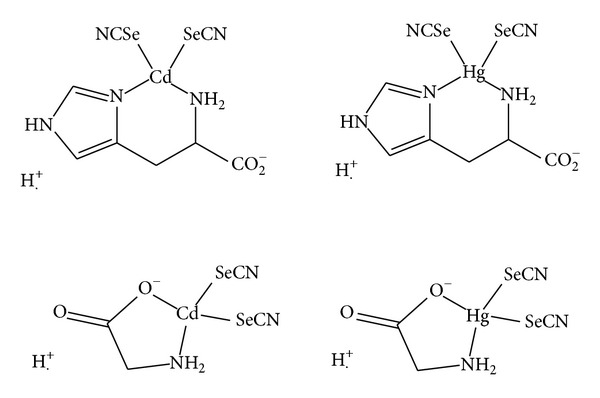
Possible binding sites for Cd^2+^ and Hg^2+^ His and Gly complexes.

The CPMAS NMR spectral data for complexes (Gly)Cd(SeCN)_2_ and (His)Cd(SeCN)_2_ for ^13^C and (Gly)Hg(SeCN)_2_ and (His)Hg(SeCN)_2_ for ^15^N are shown in Tables 5 and 6, respectively. The solid-state ^13^C and ^15^N NMR spectra are shown in Figures 1 and 2, and the peaks are denoted by asterisk. The calculated chemical shift tensors are also compiled in Tables 5 and 6, along with the span, Ω, which describes the breadth of the chemical shift tensor and skew, *κ*, describing the shape of the powder pattern. From Table 5, solid-state ^13^C NMR of Glycine and Histidine cadmium complexes shows increase in the chemical of the Se^13^CN NMR shift increased by ~2 ppm for mercury complex this is because the involvement of selenium in binding to the metal center, causing deshielding at the SeCN carbon. But in case of the ^15^N NMR data in Table 6, Histidine and Glycine complexes show a significant downfield shift of the nitrogen atom signal of the amines group; additionally, Histidine shows downfield shift for the imidazole moiety, which improves imidazole nitrogen involvement in binding to metal.

### 3.2. Computation Study

**Scheme 1:**
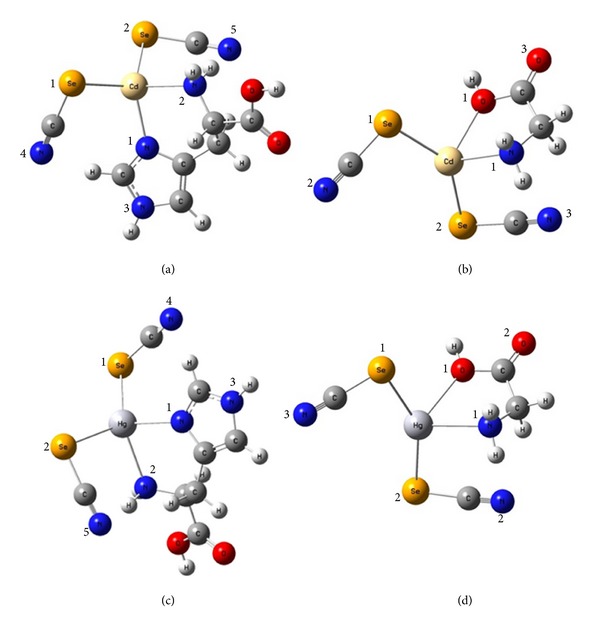
Optimized geometries of [LM(SeCN)_2_] (a), (b), (c), and (d), obtained at the B3LYP/LanL2DZ level of theory using Gaussian 09, Revision A. 1. L refers to Histidine or Glycine, while M refers to Hg or Cd.

Computational study shows that Glycine (C=O) and (C–O) bonds in the optimized structure (Scheme 1) are shorter in Cd complex than that in Hg complex (Tables 7 and 8), which agree with experimental ^13^C NMR results, while no differences in (C=O) and (C–O) bonds were observed in His complexes indicating less contribution of carboxylate in binding. Se–Cd is shorter than Se–Hg because of the size proximity in the sizes of Se and Cd atoms. It is worth mentioning here that the calculated bond lengths are comparable to reported experimental bond lengths for Se–Cd obtained by single crystals (2.723 and 2.828 Å) [20, 21]. It is also observed that Hg–N and Hg–O are longer than Cd–N and Cd–O because Cd is harder than Hg, and this results in better interaction. This may cause higher stability of Cd complexes in general. Nitrogen is a less electronegative atom than oxygen, so it can donate electron more easily to the metal and form stronger bonds with metals, which results in stronger chelation through two nitrogens than chelation through nitrogen and oxygen. L-Histidine complexes of Hg and Cd have shorter bonds than Glycine complexes indicating stronger bonds in Histidine complexes, and this agrees with the higher electron donation concluded from ^77^Se NMR data.

### 3.3. Antibacterial Activity

The *in vitro* antibacterial activity studies were performed with Cd(II) complexes against activity of both gram-positive as well as gram-negative bacteria. Two complexes, (His)Cd(SeCN)_2_ and (Gly)Cd(SeCN)_2_, exhibited their antibacterial activity compared to Cd(SeCN)_2_ except for *K. pneumoniae* which showed resistance to all the compounds tested, and Hg(SeCN)_2_ inhibition was reported previously [15], while mercury complexes with Glycine and Histidine did not show significant antibacterial activity. The activities of the complexes are summarized in Table 9.

## 4. Conclusions

We have described the synthesis of Cd(II) and Hg(II) complexes of the type, [(L)M(SeCN)_2_] (where L=Histidine or Glycine and M=Cd^2+^ or Hg^2+^), for use as a potential antibacterial agents. Characterization of these compounds by EA, IR solution, and solid NMR of various nucleuses reveals that the metal complexes with Histidine are more strongly coordinated than that of the corresponding Glycine containing metal complexes. The Cd(II) complexes have shown good zone inhibition towards different microorganisms, and thier further biological evaluation is under process, while no significant antibacterial activity was observed for the mercury complexes.

## Figures and Tables

**Table 1:** Elemental analysis of the prepared complexes.

Complex	M. Pt. (C)	Found (Calcd.)%		
		H	C	N
(Gly)Cd(SeCN)_2_	Decomp. at 184	1.32 (1.27)	12.15 (12.09)	10.78 (10.57)
(Gly)Hg(SeCN)_2_	>300	1.10 (1.04)	10.04 (9.89)	9.00 (8.65)
(His)Cd(SeCN)_2_	Decomp. > 205	2.00 (1.90)	20.44 (20.12)	14.88 (14.67)
(His)Hg(SeCN)_2_	Decomp. > 140	1.70 (1.60)	17.11 (16.99)	12.64 (12.38)

**Table 2:** IR frequencies, *ν*(cm^−1^) Hg(SeCN)_2_ and Cd(SeCN)_2_ complexes theoretical versus experimental.

Species	*ν*(C=O) Exp.	*ν*(C=O) Theo.	*ν*(SeCN) Exp.	*ν*(SeCN) Theo.	*ν*(NH_2_) Exp.	*ν*(NH_2_) Theo.
KSeCN	—	—	2070^a^	—	—	—
L-Gly	1606 s	—	—	—	3424	—
L-Hist	1634 s	—	—	—	3127	—
Cd(SeCN)_2_	—	—	2107	—	—	—
(L-Gly)Cd(SeCN)_2_	1611 s	1759	2107	2127	3450	3455
(L-Hist)Cd(SeCN)_2_	1631 s	1718	2109	2112	3460	3402
Hg(SeCN)_2_	—	—	2127	—	—	—
(L-Gly)Hg(SeCN)_2_	1611 s	1742	2130	2137	3447	3476
(L-Hist)Hg(SeCN)_2_	1636 s	1716	2111	2118	3422	3423

^a^[14].

**Table 3:** ^
13^C NMR chemical shifts of Hg(SeCN)_2_ and Cd(SeCN)_2_ complexes in DMSO-*d*_6_.

Species	SeCN	C=O	C-1	C-2	C-3	C-4	C-5
His	—	174.7	136.2	135.0	117.9	55.1	29.0
Gly	—	173.1	42.5				
Cd(SeCN)_2_	116.9						
(His)Cd(SeCN)_2_	115.4	173.0	136.6	134.5	117.0	53.5	28.1
(Gly)Cd(SeCN)_2_	119.0	194.9					
Hg(SeCN)_2_	103.3						
(His)Hg(SeCN)_2_	109.8	170.3	135.0	132.3	116.4	53.3	27.4
(Gly)Hg(SeCN)_2_	116.5	189.2					

**Table 4:** ^
77^Se NMR chemical shifts of Hg(SeCN)_2_ and Cd(SeCN)_2_ complexes in DMSO-*d*_6_.

Species	^ 77^Se (in ppm)
Cd(SeCN)_2_	−272.94
Hg(SeCN)_2_	−109.18
(His)Hg(SeCN)_2_	−169.71

**Table 5:** Solid-state ^13^C Isotropic Chemical Shifts (*δ*_iso_) and Principle Shielding Tensors(*σ*_*xx*_)^a ^ of complexes Cd(II)-Selenocyanate complexes with Glycine and Histidine ligands.

Complex	Nucleus	*δ*_iso_	*σ*_11_	*σ*_22_	*σ*_33_	Δ*σ*	*η*
Cd(SeCN)_2_	^ 113^Cd	211.9	322	283	30	291	0.73
	^ 77^Se	−119.6	53	41	−452	505	0.96
	^ 13^C	117.0	222	205	−76	298	0.89

(Gly)Cd(SeCN)_2_	^ 13^C	170.8	242	171	98	−109	0.98
	^ 13^C	119.9	212	124	23	−146	0.90

(His)Cd(SeCN)_2_	^ 13^C	169.3	236	169	102	−101	0.99
	^ 13^C	108.4	181	103	41	−101	0.86
	^ 13^C	132.0	202	136	58	−111	0.90
	^ 13^C	129.2	196	130	61	−102	0.96
	^ 13^C	119.5	213	120	23	−142	0.97

^a^Isotropic shielding,*σ*_*i*_ = (*σ*_11_ + *σ*_22_ + *σ*_33_)/3; Δ*σ* = *σ*_33_ − 0.5 (*σ*_11_ + *σ*_22_); *η* = 3(*σ*_22_ − *σ*_11_)/2Δ*σ*.

**Table 6:** Solid-state ^15^N isotropic chemical shifts (*δ*_iso_) and principle shielding tensors (*δ*_*xx*_)^a ^ of complexes, Hg(II)-selenocyanate complexes.

Complex	Nucleus	*δ*_iso_	*δ*_11_	*δ*_22_	*δ*_33_	Δ*σ*	*η*
His	^ 15^N	−202.55	−97.76	−181	−328.85	−189.45	0.66
	^ 15^N	−331.02	—	—	—	—	—
(His)Hg(SeCN)_2_	^ 15^N	−156.73	−27.66	—	−272.80	−174.11	0.80
	^ 15^N	−146.5	—	169.72	—	—	—
Gly	^ 15^N	−345.56	—	—	—	—	—
(Gly)Hg(SeCN)_2_	^ 15^N	−311.01	—	—	—	—	—

^a^Isotropic shielding, *σ*_*i*_: (*σ*_11_ + *σ*_22_ + *σ*_33_)/3; Δ*σ*: *σ*_33_ − 0.5(*σ*_11_ + *σ*_22_); *η*: 3(*σ*_22_ − *σ*_11_)/2Δ*σ*.

**Table 7:** Selected bond lengths (Å) for [LM(SeCN)_2_] for optimized structure using B3LYP/LanL2DZ; L refers to Histidine and Glycine, while M refers to Hg or Cd.

Hg(SeCN)_2_ + His		Cd(SeCN)_2_ + His		Hg(SeCN)_2_ + Gly		Cd(SeCN)_2_ + Gly	
Hg-Se1	2.765	Cd-Se1	2.718	Hg-Se1	2.699	Cd-Se1	2.656
Hg-Se2	2.795	Cd-Se2	2.745	Hg-Se2	2.731	Cd-Se2	2.691
Hg-N1	2.434	Cd-N1	2.302	Hg-N1	2.576	Cd-N1	2.415
Hg-N2	2.537	Cd-N2	2.399	Hg-O1	2.656	Cd-O1	2.423
C=O	1.236	C=O	1.236	C=O	1.232	C=O	1.228
C–O	1.386	C–O	1.386	C–O	1.402	C–O	1.412

**Table 8:** Selected torsion angle (°) for [LM(SeCN)_2_] for optimized structure using B3LYP/LanL2DZ; L refers to Histidine and Glycine, while M refers to Hg or Cd.

Hg(SeCN)_2_ + His		Cd(SeCN)_2_ + His		Hg(SeCN)_2_ + Gly		Cd(SeCN)_2_ + Gly	
Se1-C-N4	178.79	Se1-C-N4	178.45	Se1-C-N3	178.03	Se1-C-N2	178.20
Se2-C-N5	176.36	Se2-C-N5	175.84	Se2-C-N2	177.04	Se2-C-N3	176.32
N1-Hg-N2	83.87	N1-Cd-N2	87.79	N1-Hg-O1	64.67	N1-Cd-O1	96.81
Se1-Hg-Se2	125.79	Se1-Cd-Se2	121.32	Se1-Hg-Se2	149.92	Se1-Cd-Se2	139.71
Se1-Hg-N1	108.92	Se1-Cd-N1	111.15	Se1-Hg-N1	107.50	Se1-Cd-N1	112.35
Se2-Hg-N2	99.18	Se2-Cd-N2	101.26	Se2-Hg-O1	111.58	Se2-Cd-O1	112.17
Se1-Hg-N2	118.69	Se1-Cd-N2	116.85	Se1-Hg-O1	89.99	Se1-Cd-O1	96.81
Se2-Hg-N1	112.60	Se2-Cd-N1	113.30	Se2-Hg-N1	100.81	Se2-Cd-N1	103.86

**Table 9:** Antibacterial activities of [LM(SeCN)_2_] complexes.

Microorganisms	Zone of inhibition (mm)			
	Hg(SeCN)_2_*	Cd(SeCN)_2_	(His)Cd(SeCN)_2_	(Gly)Cd(SeCN)_2_
*E. coli *	—	25	35	22
*P. aeruginosa *	10	20	18	32
*S. typhi *	10	28	32	29
*S. aureus *	22	20	22	20
*K. pneumoniae *	22	—	—	—

*[15]

## References

[1] Keene FR, Smith JA, Collins JG (2009). Metal complexes as structure-selective binding agents for nucleic acids. *Coordination Chemistry Reviews*.

[2] Drewry JA, Gunning PT (2011). Recent advances in biosensory and medicinal therapeutic applications of zinc(II) and copper(II) coordination complexes. *Coordination Chemistry Reviews*.

[3] Marino T, Russo N, Toscano M (2001). Potential energy surfaces for the gas-phase interaction between *α*-alanine and alkali metal ions (Li^+^, Na^+^, K^+^). A density functional study. *Inorganic Chemistry*.

[4] Orvig C, Abrams MJ (1999). Medicinal inorganic chemistry: introduction. *Chemical Reviews*.

[5] Guo Z, Sadler PJ (1999). Medicinal inorganic chemistry. *Advances in Inorganic Chemistry*.

[6] Bruijnincx PC, Sadler PJ (2008). New trends for metal complexes with anticancer activity. *Current Opinion in Chemical Biology*.

[7] Peschke M, Blades AT, Kebarle P (2000). Metalloion-ligand binding energies and biological function of metalloenzymes such as carbonic anhydrase. A study based on ab initio calculations and experimental ion-ligand equilibria in the gas phase. *Journal of the American Chemical Society*.

[8] Marino T, Russo N, Toscano M (2000). Gas-phase metal ion (Li^+^, Na^+^, Cu^+^) affinities of glycine and alanine. *Journal of Inorganic Biochemistry*.

[9] Marino T, Toscano M, Russo N, Grannd A (2006). Structural and electronic characterization of the complexes obtained by the interaction between bare and hydrated first-row transition-metal ions (Mn^2+^, Fe^2+^, Co^2+^, Ni^2+^, Cu^2+^, Zn^2+^) and glycine. *The Journal of Physical Chemistry B*.

[10] Ai H, Bu Y, Han K (2003). Glycine-Zn^+^/Zn^2+^ and their hydrates: on the number of water molecules necessary to stabilize the switterionic glycine-Zn^+^/Zn^2+^ over the nonzwitterionic ones. *Journal of Chemical Physics*.

[11] Pesonen H, Sillanpaä A, Aksela R, Laasonen K (2005). Density functional complexation study of metal ions with poly(carboxylic acid) ligands—part 1: poly(acrylic acid) and poly(*α*-hydroxy acrylic acid). *Polymer*.

[12] Hasegawa K, Ono T, Noguchi T (2002). Ab initio density functional theory calculations and vibrational analysis of zinc-bound 4-methylimidazole as a model of a histidine ligand in metalloenzymes. *Journal of Physical Chemistry A*.

[13] Leary JA, Zhou Z, Ogden SA, Williams TD (1990). Investigations of gas-phase lithium-peptide adducts: tandem. mass spectrometry and semiempirical studies. *Journal of the American Society for Mass Spectrometry*.

[14] Li SL, Wu JY, Tian YP (2006). Design, crystal growth, characterization, and second-order nonlinear optical properties of two new three-dimensional coordination polymers containing selenocyanate ligands. *European Journal of Inorganic Chemistry*.

[15] Nasiruzzaman Shaikh M, Al-Maythalony BA, Wazeer MIM, Isab AA (2011). Complexations of 2-thiouracil and 2,4-dithiouracil with Cd(SeCN)_2_ and Hg(SeCN)_2_: NMR and anti-bacterial activity studies. *Spectroscopy*.

[16] Barraud P, Schubert M, Allain FHT (2012). A strong ^13^C chemical shift signature provides the coordination mode of histidines in zinc-binding proteins. *Journal of Biomolecular NMR*.

[17] Wazeer MIM, Isab AA, Fettouhi M (2007). New cadmium chloride complexes with imidazolidine-2-thione and its derivatives: X-ray structures, solid state and solution NMR and antimicrobial activity studies. *Polyhedron*.

[18] Boeckmann J, Reinert T, Näther C (2011). Investigations on the thermal reactivity of Zn^II^ and Cd^II^ selenocyanato coordination compounds. *Zeitschrift für Anorganische und Allgemeine Chemie*.

[19] Shukla SN, Gaur P, Mathews S, Khan S, Srivastava A (2008). Synthesis, characterization, catalytic and biological activity of some bimetallic selenocyanate Lewis acid derivatives of *N* , N′- *bis* (2-chlorobenzylidene)ethylenediamine. *Journal of Coordination Chemistry*.

[20] Sain S, Maji TK, Mostafa G, Lu TH, Chiang MY, Chaudhuri NR (2002). Self assembly towards the construction of molecular ladder and rectangular grid of cadmium(II)-selenocyanate. *Polyhedron*.

[21] Maji TK, Sain S, Mostafa G, Das D, Lu TH, Chaudhuri NR (2001). Synthesis and crystal structure of selenocyanato bridged two dimensional supramolecular coordination compounds of cadmium(II). *Journal of the Chemical Society, Dalton Transactions*.

[22] Isab AA, Wazeer MIM (2005). Complexation of Zn(II), Cd(II) and Hg(II) with thiourea and selenourea: a ^1^H, ^13^C, ^15^N, ^77^Se and ^113^Cd solution and solid-state NMR study. *Journal of Coordination Chemistry*.

[23] Hayashi S, Hayamizu K (1991). Chemical shift standards in high-resolution solid-state NMR (2) ^15^N nuclei. *Bulletin of the Chemical Society of Japan*.

[24] Kemp W (1986). *NMR in Chemistry*.

[25] Eichele K, Wasylischen RE (2001). *W: Simulation Package, Version 1. 4. 4*.

[26] Colaneri MJ, Peisach J (1995). A single crystal EPR and ESEEM analysis of Cu(II)-doped bis(L-histidinato)cadmium dihydrate. *Journal of the American Chemical Society*.

[27] Zhanpeisov NU, Matsuoka M, Yamashita H, Anpo M (1998). Cluster quantum chemical ab initio study on the interaction of NO molecules with highly dispersed titanium oxides incorporated into silicalite and zeolites. *Journal of Physical Chemistry B*.

[28] Nicklass A, Dolg M, Stoll H, Preuss H (1995). Ab initio energy-adjusted pseudopotentials for the noble gases Ne through Xe: calculation of atomic dipole and quadrupole polarizabilities. *The Journal of Chemical Physics*.

[29] Frisch M, Trucks G, Schlegel H (2009). *Gaussian 09, Rev. A. 1*.

[30] Navarro V, Villarreal ML, Rojas G, Lozoya X (1996). Antimicrobial evaluation of some plants used in Mexican traditional medicine for the treatment of infectious diseases. *Journal of Ethnopharmacology*.

[31] Brooks P, Davidson N (1960). Mercury (II) complexes of imidazole and histidine. *Journal of the American Chemical Society*.

[32] Renuka A, Shakuntala K, Srinivasan PC (1998). *Indian Journal of Chemistry*.

